# Short-Term Exposure to Ambient Air Pollution and Hospital Admission for Stroke: A Multi-Center Hospital-Based Case-Crossover Study

**DOI:** 10.3390/toxics14080685

**Published:** 2026-08-03

**Authors:** Xiaona Li, Rui Wang, Ruijun Xu, Ling Deng, Sijia Wang, Kui Xie, Qiuying Li, Xuan Luo, Yuewei Liu, Wancheng Ma

**Affiliations:** 1 Department of Non-Communicable Disease Prevention and Control, Luohu Center for Chronic Disease Control, Shenzhen 518000, China; li15066288873@163.com (X.L.); wangrui.1983@hotmail.com (R.W.); 15219711192@163.com (L.D.); wangsj26@mail3.sysu.edu.cn (S.W.); 13530200683@163.com (K.X.); l999qy@163.com (Q.L.); luox225@alumni.sysu.edu.cn (X.L.); 2 Department of Epidemiology, School of Public Health, Sun Yat-sen University, Guangzhou 510080, China; xurj25@mail.sysu.edu.cn

**Keywords:** ambient air pollution, stroke admission, time-stratified case-crossover study

## Abstract

Stroke is a leading cause of death and disability worldwide. Exposure to ambient air pollutants and the risk of stroke have been reported in many studies, but the results vary greatly among different regions. This study aims to investigate the association between short-term exposure to air pollution and the admission of total stroke and its subtypes in the Luohu District, Shenzhen. A time-stratified case-crossover study was conducted among 21,973 newly diagnosed stroke cases in Luohu, Shenzhen from 2014 to 2022. Residential exposure to air pollution was assessed using validated grid datasets. A distributed lag model (DLM) and conditional logistic regression model were implemented to evaluate the relationship between ambient air pollution and the admission of stroke and its subtypes. We found that a 10 µg/m^3^ increase in exposure to NO_2_ and SO_2_ was positively associated with a 2.73% increase (95% confidence interval [CI]: 2.21%, 3.25%) and a 24.89% increase (20.87%, 29.06%) in the odds of total stroke admission, respectively. Statistical significance has also been found in subtypes. Stronger associations were observed in females (SO_2_), the elderly (NO_2_ and SO_2_), and the cold season (NO_2_ and SO_2_). Our findings indicate that exposure to NO_2_ and SO_2_ exacerbates the risk of stroke admission, especially in the elderly, females, and the cold season.

## 1. Introduction

Stroke is a prevalent neurological disease caused by insufficient cerebral blood supply (ischemic stroke) or a loss of vascular integrity leading to bleeding in the brain parenchyma (hemorrhagic stroke), which can result in death and disability in severe cases [1,2]. The number of deaths and disabilities caused by stroke has rapidly increased over the past 30 years. According to the Global Burden of Disease Study 2023 [3,4], stroke remained the second cause of death and the third cause of disability-adjusted life-years (DALYs) among non-communicable disorders (NCDs) globally. Given that the total number of stroke-related DALYs due to risk factors increased substantially from 1990 (100 million) to 2023 (157 million), understanding risk factors has become a priority in making prevention and control measures for stroke.

Previous studies have demonstrated that air pollution exposure may increase the risk of stroke. A systematic review and meta-analysis with a total of 6.2 million stroke events in 28 countries reported that an increase in concentrations of carbon monoxide (CO), sulfur dioxide (SO_2_), nitrogen dioxide (NO_2_), fine particle (PM_2.5_), inhalable particle (PM_10_), and ozone (O_3_) was related to admission to hospital for stroke or mortality from stroke [5]. However, this review only reported the effect of short-term exposure to air pollutants on total stroke. In terms of stroke subtypes, hemorrhagic stroke events are less than ischemic stroke events, resulting in reduced statistical power and fewer studies that examined it as a distinct outcome. The effects of air pollutants on stroke subtypes vary greatly in different regions and countries. For example, in South London, Butland et al. [6] found no evidence that exposure to PM_2.5_, PM_10_, O_3_ or NO_2_ was associated with ischemic stroke. For hemorrhagic stroke, they observed an inverse correlation with PM_10_. Researchers have found a positive relationship between hemorrhagic stroke admission and exposure to PM_2.5_ in Taiwan [7].

Therefore, further research is needed to analyze the impact of air pollutants from different regions or countries on the total stroke and its different subtypes (such as ischemic and hemorrhagic stroke) among residents in those regions. This study employs a time-stratified case-crossover research method to examine the effects of short-term exposure to gaseous and particulate pollutants on first admissions for stroke and its subtypes in the Luohu District, Shenzhen. We also conducted stratified analyses to detect potential effects.

## 2. Materials and Methods

### 2.1. Study Population

The diagnostic criteria for stroke are based on the Atherosclerosis Risk in Communities (ARIC); they have been described in a previous study [8]. We collected data on 31,507 stroke patients residing in Luohu District from daily stroke admission records of general hospitals in Shenzhen from 1 January 2014 to 31 December 2022. Individual data on age, sex, date of admission, the International Statistical Classification of Diseases and Related Health Problems, 10th Revision (ICD-10), number of episodes, and residential address were included for each subject. Based on the number of episodes recorded by the hospital, we excluded 9534 patients with a history of stroke and incomplete data. In this study, we included 21,973 newly diagnosed stroke patients, including hemorrhagic stroke (ICD-10 code: I60-62), ischemic stroke (ICD-10 code: I63), and unspecified stroke (ICD-10 code: I64).

### 2.2. Study Design

A time-stratified case-crossover design was used to assess the association of air pollution exposure and stroke hospital admission [9]. This design has been widely used to explore the relationship of exposure to air pollutants and health conditions [10,11]. In this design, we defined the date of admission as the case day and chose the same day in the other weeks of the same year and month (the time stratum) as the control day. For instance, if a subject’s admission time is 7 July 2022 (Thursday), then 7 July 2022 was defined as the case day, and all other Thursdays in July 2022 (i.e., 14, 21, and 28 July) were defined as the corresponding control days. According to this method, we matched 74,832 control days for 21,973 case days in our final analysis. This design could control the impacts of time-invariant variables, long-term trends, and seasonality [12].

### 2.3. Exposure Assessment

Daily grid data (spatial resolution: 1 km × 1 km) on daily 24-h average PM_2.5_, PM_10_, SO_2_, NO_2_, CO, and daily peak 8-h average O_3_ concentrations in Luohu during 2014–2022 were retrieved from the ChinaHighAirPollutant (CHAP) dataset (accessible at: https://weijing-rs.github.io/product.html; accessed on 15 October 2024). The dataset was generated using a comprehensive national ground observation network, big data, and artificial intelligence technologies, with comprehensive coverage, high resolution, and excellent quality [13]. The cross-validated coefficient of determination (R^2^) for PM_2.5_, PM_10_, SO_2_, NO_2_, CO, and O_3_ was 0.92, 0.90, 0.84, 0.93, 0.80, and 0.92, respectively [13,14,15,16,17]. Exposure data at each subject’s geocoded residential address were extracted using the bilinear interpolation method. As proposed in previous studies, the air pollution exposure metric was defined as the case and control days and the preceding 3 days.

### 2.4. Covariates

Daily 24 h average temperature (°C) and relative humidity (%) were obtained from the China Meteorological Administration Land Data Assimilation System (CLDAS version 3.0; spatial resolution: 0.0625° × 0.0625°) [18]. We calculated HI using the following formulas:

(1) If $T_{a}$ ≤ 40 °F:

(1) $$\mathrm{HI}\;=\;T_{a},$$

(2) If $T_{a}$ > 40 °F:

(3) If $T_{a}$ > 40 °F and HI < 79 °F:

(2) $$\mathrm{HI}\;=\;-10.3\;+\;1.1\;\times \;T_{a}\;+\;0.047\;\times \;\mathrm{RH},$$

(4) If HI ≥ 79 °F, the following formula was used with adjustments for certain relative humidity ranges:

(3) $$\begin{array}{ll} HI=-42.379 & +\;2.04901523\times T_{a}+10.14333127\times RH\\ & \;\;-0.22475541\times T_{a}\times RH-6.83783\times 10^{-3}\times T_{a}^{2}\\ & \;\;-5.481717\times 10^{-2}\times {RH}^{2}+1.22874\times 10^{-3}\times T_{a}^{2}\times RH\\ & \;\;+8.5282\times 10^{-4}\times T_{a}\times {RH}^{2}-1.99\times 10^{-6}\times T_{a}^{2}\times {RH}^{2}, \end{array}$$

(5) If RH ≤ 13% and 80 °F ≤ $T_{a}$ ≤ 112 °F:

(4) $$HI=HI-\frac{13-RH}{4}\times \sqrt{\frac{17-\left|T_{a}-95\right|}{17}},$$

(6) If RH > 85% and 80 °F ≤ $T_{a}$ ≤ 87 °F:

(5) $$HI=HI+0.02\times \left(RH-85\right)\times \left(87-T_{a}\right),$$

where HI is heat index (°F); $T_{a}$ is daily air temperature (°F); RH is daily relative humidity (%).

### 2.5. Statistical Analysis

Continuous variables were described using mean and standard deviation (SD), while categorical variables were described using number and percentage. A *t*-test was used to compare whether pollutant concentrations differed between case days and control days. Spearman’s correlation coefficient was used to estimate correlations between air pollutants.

A conditional logistic regression model and a distributed lag model (DLM) were employed to explore the correlation of air pollution exposure and the odds of total stroke and cause-specific stroke admissions, respectively. The model is as follows:

(6) Logit (Pt)=cb (Xt,lag)+cb (HI,lag)+α_i,

where t is day of observation, Pt refers to the probability of stroke occurring on the t day, cb (Xt, lag) and cb (HI, lag) indicate the matrix of air pollution and heat index by applying the DLM (exposure was set as linear function) or DLNM (exposure was set as natural cubic spline function), Xt and HI represent pollutant concentrations and heat index at day t. The primary models incorporated natural cubic spline functions with 3 degrees of freedom (*df*) for HI [19,20], as well as a DLM-based cross-basis function for air pollution exposure [21]. A linear function for the space of air pollution exposures and the space of 4-day lag form the cross-basis function [21,22]. The percentage change in odds of stroke admission (calculated as [odds ratio − 1] × 100%) and its 95% confidence interval (CI) were calculated to quantify the cumulative associations for exposure to air pollution in lag 0–3 for each 10 µg/m^3^ increment of PM_2.5_, PM_10_, SO_2_, NO_2_, O_3_, and each 1 mg/m^3^ of CO exposures. To assess whether the relationship was non-linear, likelihood ratio tests were used. Additionally, we used distributed lag non-linear models (DLNMs), where the space of air pollution exposures uses a cross-basis function with a natural cubic spline function (*df* = 3), while the space of 4-day lag uses a linear function. We used the lowest air pollution exposure level as the reference to evaluate the relationship between exposure to air pollution and admissions for total and cause-specific stroke. We also divided the concentration of pollutants into low and high groups based on the median; the low-concentration group served as the reference group to analyze the impact of pollutants in the high-concentration group on stroke. To avoid multicollinearity, we only adjusted the meteorological variable HI, which combines meteorological variables such as temperature and humidity.

Stratification analyses were conducted by sex (male, female), age (<65 years, ≥65 years), and season (warm and cold) to explore potential effects. The 2-sample z test was used to examine the difference in risk estimates for each stratification subgroup, with the formula as follows:

(7) $$z=\frac{\beta_{1}-\beta_{2}}{\sqrt{{SE}_{\beta_{1}}^{2}+{SE}_{\beta_{2}}^{2}}},$$

where β represents the stratification-specific point estimates (i.e., ln odds ratio); SE represents the standard error.

Sensitivity analyses were conducted to evaluate the robustness of the associations between the six air pollutants and stroke. In the sensitivity analysis, we conducted 2-pollutant models (Correlation coefficient r > 0.70 between air pollutants was not included in the multipollutant model to avoid collinearity), in which each air pollutant was additionally adjusted for another pollutant in the same model. R software (version 4.3.3; Vienna, Austria) was used for all the analyses. The statistical tests were two-sided, and *p* < 0.05 was considered statistically significant.

## 3. Results

### 3.1. Descriptive Results

A total of 21,973 subjects were included in this study. Among them, 59.0% were males, 53.6% were under 65 years old, and 81.5% of the subjects were diagnosed with ischemic stroke (Table 1). The mean concentration of CO, NO_2_, O_3_, PM_10_, PM_2.5_ and SO_2_ on the case day was 0.76 mg/m^3^, 31.37 µg/m^3^, 91.50 µg/m^3^, 43.39 µg/m^3^, 25.31 µg/m^3^ and 8.03 µg/m^3^, respectively. The pollutant concentration between the case day and the control day showed no significant difference. The mean HI on the case days was 25.63 °C (range: 3.84 °C to 45.02 °C) (Table 2). Spearman’s correlation analyses show that all air pollutants were intercorrelated, and the correlations between PM_2.5_, PM_10_, SO_2_, and NO_2_ ranged from moderate to strong, with a correlation coefficient (r) higher than 0.50 (Table 3).

### 3.2. Associations Between Air Pollution and Stroke

The associations between exposure to air pollution and stroke admission were shown in Table 4. In single-pollutant models, CO, NO_2_, PM_10_, PM_2.5_, and SO_2_ exposure was significantly associated with increased risks of total stroke admission. The cumulative associations from lag 0 to lag 3 were observed on CO, NO_2_, PM_10_, PM_2.5_ and SO_2_ with the increased risk of 6.98% (2.61%, 11.53% for each 1 mg/m^3^ increasement), 2.73% (2.21%, 3.25% for each 10 µg/m^3^ increasement), 1.26% (1.00%, 1.52% for each 10 µg/m^3^ increasement), 1.87% (95% CI: 1.37%, 2.38% for each 10 µg/m^3^ increasement) and 24.89% (20.87%, 29.06% for each 10 µg/m^3^ increasement), respectively. No significant correlation was found between O_3_ and total stroke admission.

**Figure 1:**
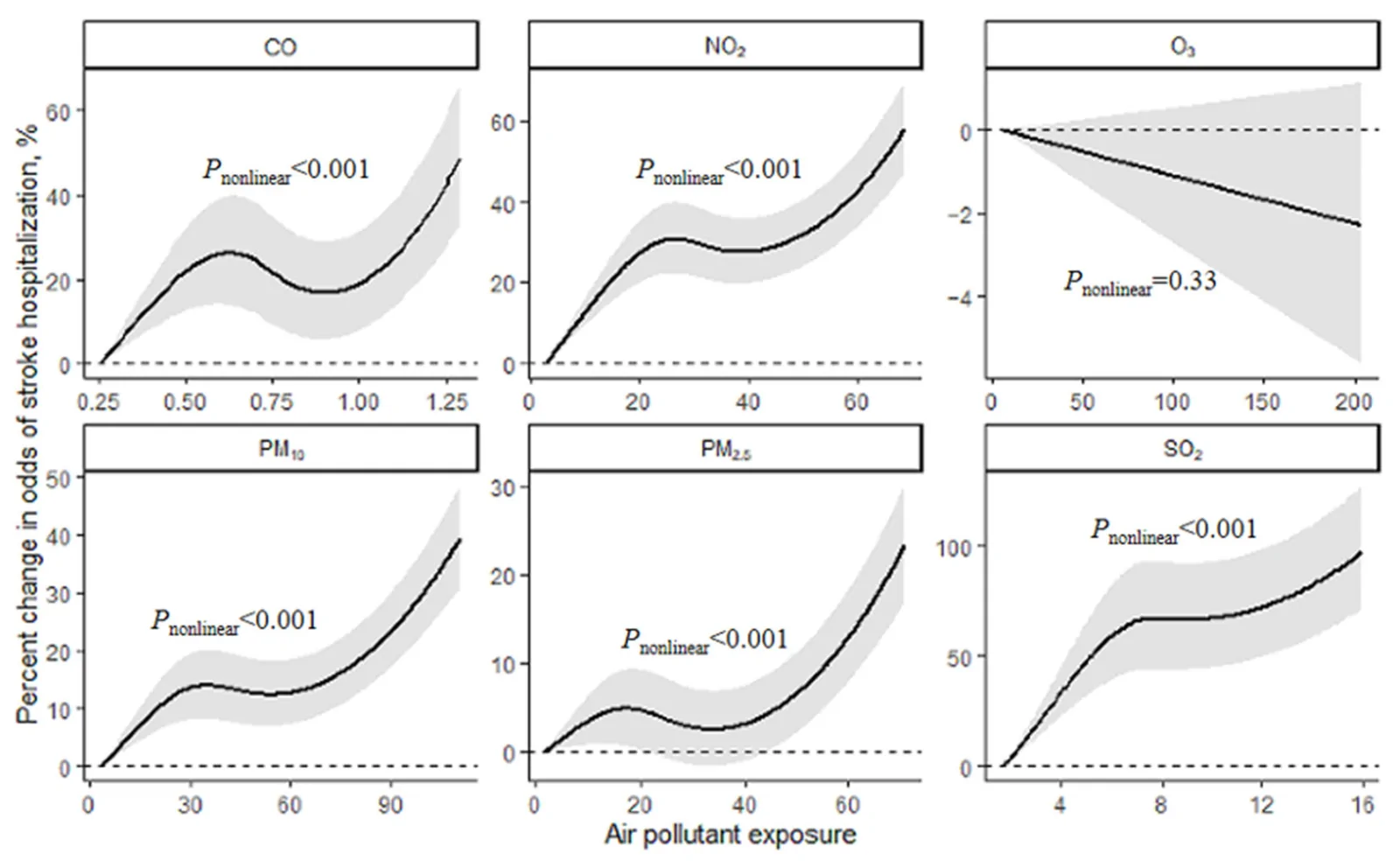
Cumulative exposure-response curves for associations of lag 0–3 days exposure to air pollution with admission from total stroke. The horizontal dashed line represents the percent change of 0.

The association of CO, NO_2_, PM_10_, PM_2.5_, and SO_2_ exposure with stroke admission was nonlinear (*p* for nonlinearity < 0.001; Figure 1). The odds of stroke hospitalization increased with lower CO, NO_2_, PM_10_, PM_2.5_, and SO_2_ exposure. At concentrations of approximately 0.6 mg/m^3^, 27 µg/m^3^, 36 µg/m^3^, 20 µg/m^3^, and 8 µg/m^3^, the odds of stroke hospitalization decreased with increasing pollution concentration, while at concentrations of about 1 mg/m^3^, 40 µg/m^3^, 55 µg/m^3^, 35 µg/m^3^, and 40 µg/m^3^, the odds of stroke hospitalization increased with increasing pollutant concentration. The association of O_3_ exposure with stroke admission was linear (*p* = 0.33). Compared with low groups, high groups of NO_2_ and SO_2_ had a positive association with total stroke admission (Table A1).

**Figure 2:**
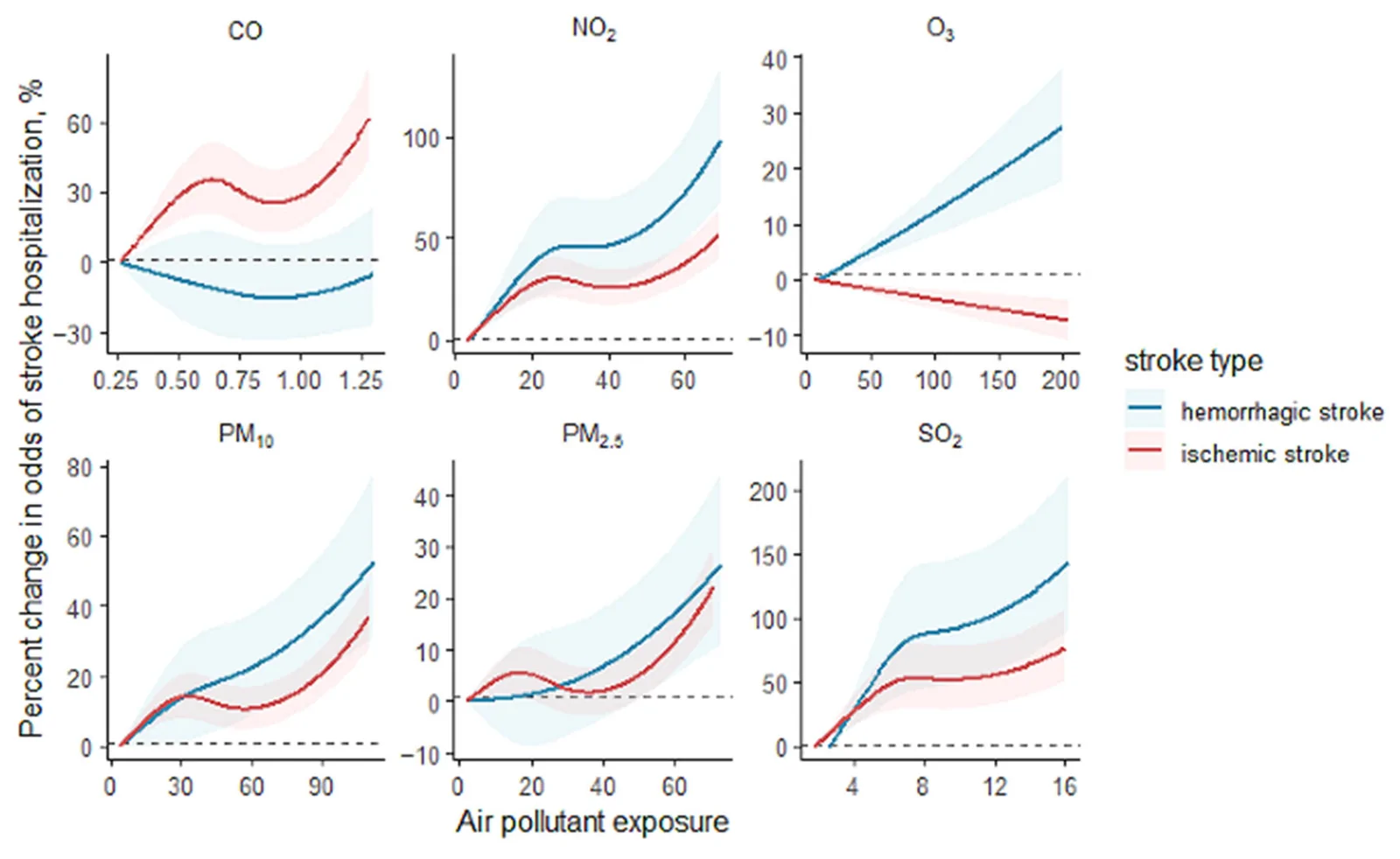
Cumulative exposure-response curves for associations of lag 0–3 days exposure to air pollution with admission from hemorrhagic stroke and ischemic stroke. The horizontal dashed line represents the percent change of 0.

The associations between ischemic and hemorrhagic stroke and air pollutant exposure showed that NO_2_, PM_10_, PM_2.5_, and SO_2_ had significant effects on both types of strokes (Table 4, Figure 2). O_3_ could decrease the odds of ischemic stroke and hemorrhagic stroke admission. CO only has a significant effect on ischemic stroke, with the percent change in ischemic stroke for every 1 mg/m^3^ increase in concentrations of CO being 8.49% (95% CI: 3.61%, 13.61%) in lag 0–3.

### 3.3. Stratified Analysis and Sensitivity Analysis

The relationship between air pollution exposures and stroke admission stratified by sex, age, and season is shown in Table 5. Gender stratification showed that NO_2_, PM_10_, PM_2.5_, and SO_2_ increased the odds of stroke hospital admission in men and women, but the effect of SO_2_ on women was significantly higher than that of men (*p* = 0.03). CO is only related to an increased hospital admission of stroke in men, with an increased risk of 12.47% (95% CI: 6.55%, 18.73%) for each 1 mg/m^3^ increase in exposure. Age stratification showed that significantly harmful effects of NO_2_, PM_10_, PM_2.5_, and SO_2_ exposure on stroke admission among the elderly (≥65 years). Season stratification showed that CO, NO_2_, PM_10_, PM_2.5_, and SO_2_ exposure increased the odds of stroke hospitalization in cold seasons. The 2-pollutant analyses revealed that these associations were significant and stable in NO_2_ and SO_2_, while the associations for PM_10_, PM_2.5_, and CO became insignificant when certain other pollutants were included in the same model (Table 6).

## 4. Discussion

In this case-crossover study, we used a conditional logistic regression model and distributed lag model to explore the impact of short-term air pollutant accumulation lag on stroke admission. This study showed that NO_2_ and SO_2_ are significantly associated with increased risks of total stroke and its subtypes hospital admission. In addition, females were more susceptible to SO_2_ exposures, and NO_2_ and SO_2_ exposures had a greater impact on stroke admission in the elderly and cold season.

NO_2_ is a typical gaseous environmental air pollutant, with major sources including vehicle exhaust, fossil fuel combustion, indoor gas stoves, and cigarette smoke [23,24]. Many studies have reported the link between NO_2_ exposure and the risk of stroke admission, but the results were inconsistent. Zhou et al. [25] found an increased risk of stroke and its subtypes associated with exposure to daily NO_2_ levels in Shanghai, which is consistent with our research results. In this study, the daily concentrations of NO_2_ were 39.31 µg/m^3^, a little higher than in our study. Chen et al. [24] also reported that NO_2_ had statistically significant effects on the incidence of total stroke and ischemic stroke. However, contrary to our research findings, there was no statistically significant correlation between the increased risk of hemorrhagic stroke and ambient NO_2_ levels in Chen et al.’s study. While a large prospective cohort study conducted for women in the United States found no statistically significant association between ambient NO_2_ levels and risk of total stroke or ischemic stroke, it found a positive relationship between hemorrhagic stroke risk and NO_2_ [26]. The mean daily concentrations of air pollutants reported in these two studies were lower than the levels observed in our study area.

Similar results were also found in SO_2_ exposure and stroke admission. In the Beibu Gulf Region of China, Li et al. [27] reported that SO_2_ exposure was related to total stroke and ischemic stroke admissions. However, the association was not found in hemorrhagic stroke. Another study in Beijing also found that SO_2_ was positively associated with stroke admissions at lags 0 and 1 [28]. In these two studies, the authors evaluated the impact of single-day lag and moving-average lag exposure levels on stroke. Our study evaluated the impact of cumulative effect from lag 0 to lag 3 exposure levels on stroke. So, the associations reported between SO_2_ exposure and stroke admissions in our study are higher than in the above studies. In two Irish urban centers, Byrne et al. [29] found no significant association between all stroke admissions and SO_2_ exposure. In Chongqing, China, Chen et al. reported that SO_2_ exposure was associated with ischemic stroke in a 4-day lag, but in cumulative lag structures, this association becomes insignificant [30]. The observed inconsistencies may be attributed to differences in study population characteristics, pollutant concentration, and geographic or ethnic factors across regions [31]. The mechanism by which NO_2_ and SO_2_ increase the risk of stroke has not been fully clarified, but there is evidence that suggests inhaling these pollutants stimulates pulmonary sensory receptors, disrupting autonomic nervous system (ANS) homeostasis and activating the hypothalamic-pituitary axis [32]. ANS dysfunction is believed to cause a rapid increase in systemic blood pressure and vascular resistance, along with autonomic dysregulation, which has been consistently confirmed in controlled human exposure studies of diesel exhaust [32,33]. The acute activation of vasoconstrictor pathways and cardiac autonomic dysfunction are important mechanisms in the occurrence and development of stroke.

We also found that CO, PM_10_, and PM_2.5_ exposures were significantly related to increased risks of stroke in single-pollutant models, while this association turned insignificant in 2-pollutant models. Our research results were consistent with several recent studies. For instance, three studies done in China [34,35,36] reported significant short-term associations between CO, PM_10_ and PM_2.5_ and ischemic stroke hospitalizations in single-pollutant models. Similarly, in 2-pollutant models, the relationships for CO, PM_10_ and PM_2.5_ became non-significant when controlling for other pollution. Air pollution comprises various hazardous compounds [37,38]; given the widespread correlation among air pollutants, they may confuse with each other, which could partly account for why each pollutant showed significant effects in single-pollutant models but not in 2-pollutant models. According to the literature, another possible reason is the co-linearity issue in the regression model.

In addition, we found a negative correlation between O_3_ in the 3-day accumulation lag and ischemic stroke in single- and 2-pollutant models. It is difficult to explain from a biological perspective. A negative association has been reported in a previous study. For instance, Tian et al. [35] found that O_3_ could decrease the number of stroke patients. For every 18.7 µg/m^3^ increase in O_3_, hospital admissions for ischemic stroke decreased by 0.22% in the lag of 2 days. One possible reason is that there was a linear correlation between ozone and stroke hospitalization in our study, so the significance may be maintained even in two-pollutant models that control for other pollutants. Another reason may be related to meteorological confusion, interactions between pollutants, or exposure measurements.

Stratification by age revealed that older adults faced a higher stroke risk from ambient NO_2_ and SO_2_ exposure. This result was similar to many previous study results [25,28,35,39]. This might be because elderly individuals with compromised immunity and chronic comorbidities may be more susceptible to air-pollution exposure [24]. In our study, SO_2_ exposure showed a significantly stronger association with stroke admission in females. A study conducted in Vancouver, Canada evaluated the correlation between SO_2_ exposure and ischemic stroke and found that women have a significantly higher risk of emergency department visits for stroke than men [40]. A study in Suzhou, China also found that for every 10 µg/m^3^ increase in SO_2_ concentration, the risk of stroke in women increased by 0.88%, and no significant association was observed with men [41]. This may be due to women having smaller airways [42] with higher reactivity [43]. It means that when inhaling the same concentration of SO_2_, the burden on the female respiratory mucosa is relatively heavier. The structural difference makes pollutants more likely to cause direct damage to women’s respiratory systems, which may then affect cerebral blood vessels through systemic inflammatory reactions and other pathways. We also found that air pollution (except for O_3_) had a stronger effect on stroke in the cool season. This is consistent with most previous research findings. This is mainly because pollutants have worse diffusion in cold seasons compared to warm seasons, and low temperatures can exacerbate arterial thrombosis [44,45].

There are some strengths in our study. First, this study adopts a time-stratified case-crossover design, which can correct potential confounding factors at the individual level, long-term trends, and seasonality. Second, our study analyzed the association between air pollution and total stroke and two common subtypes, which can distinctly affect the effects of air pollution exposure and identify individuals with the highest risk. Third, the air pollution data were from the CHAP dataset, with a spatial resolution of 1 km × 1 km, which provides a more accurate assessment of individual-level exposure than studies relying solely on fixed-site monitoring stations. Nevertheless, our study has some limitations. First, we performed individual-pollutant exposure assessment based on the average pollutant concentration in the geographic area. This approach fails to adequately capture personal exposure patterns (for example, commuting, occupational exposures, cooking-related emissions, and time spent indoors). This may lead to misclassification of nondifferential exposure and may underestimate the actual effect value [46]. Second, air pollution is a complex mixture, and there is a high degree of correlation between pollutants. Although we used 2-pollutant models to adjust for the potential errors in analyzing a single-pollutant model in this study, the model still has shortcomings in evaluating the overall effects of air pollutants. Third, our subjects all come from the same region, and it is imperative to be cautious when extrapolating our findings to different populations.

## 5. Conclusions

Short-term exposure to NO_2_ and SO_2_ was significantly linked to increased risks of total and cause-specific stroke. Females are susceptible to the effects of SO_2_; exposure to SO_2_ and NO_2_ had a stronger effect in the elderly and cold season. These findings reveal the acute harmful impacts of air pollution on cerebrovascular events and emphasize the importance for both the public and policymakers to adopt effective interventions to reduce exposure to air pollution, aiding in reducing stroke admissions.

## Figures and Tables

**Table 1:** Characteristics of the study population.

Characteristic	N (%)
New cases of stroke (case days)	21,973
Control days	74,832
Age, mean ± SD	65.10 ± 14.37
<65 y	10,195 (46.4)
≥65 y	11,778 (53.6)
Sex	
Male	12,965 (59.0)
Female	9008 (41.0)
Season of onset	
Warm (May–October)	11,121 (50.6)
Cold (November–April)	10,852 (49.4)
Stroke Type	
Hemorrhagic stroke	3886 (17.7)
Ischemic stroke	17,903 (81.5)
Unspecified stroke	184 (0.8)

**Table 2:** Distribution of air pollutant exposure on case days and control days.

Variable	Mean ± SD	Min	P25	Median	P75	Max
Case days						
CO, mg/m^3^	0.76 ± 0.18	0.31	0.63	0.74	0.86	1.56
NO_2_, µg/m^3^	31.37 ± 12.66	3.04	22.53	29.44	37.81	122.15
O_3_, µg/m^3^	91.50 ± 41.42	10.02	59.13	84.87	116.71	303.78
PM_10_, µg/m^3^	43.39 ± 22.65	4.42	26.53	38.81	55.50	164.59
PM_2.5_, µg/m^3^	25.31 ± 15.15	2.21	13.87	22.33	33.29	110.45
SO_2_, µg/m^3^	8.03 ± 2.31	2.00	6.61	7.56	8.88	29.94
HI, °C	25.63 ± 7.85	3.84	19.37	25.12	32.78	45.02
Control days						
CO, mg/m^3^	0.76 ± 0.18	0.30	0.63	0.74	0.86	1.61
NO_2_, µg/m^3^	31.21 ± 12.78	3.27	22.38	29.34	37.61	120.16
O_3_, µg/m^3^	91.41 ± 41.63	7.46	58.68	84.47	116.70	307.56
PM_10_, µg/m^3^	43.10 ± 22.56	3.75	26.26	38.54	55.53	168.51
PM_2.5_, µg/m^3^	25.14 ± 14.99	1.92	13.81	22.26	33.14	114.53
SO_2_, µg/m^3^	8.01 ± 2.25	2.03	6.59	7.56	8.89	29.99
HI, °C	25.66 ± 7.94	3.74	19.30	25.28	32.88	45.41

24 h average concentration for PM_2.5_, PM_10_, SO_2_, NO_2_, and CO; maximum 8 h average concentration for O_3_. *p* for all *t*-tests between case days and control days was >0.05. CO: carbon monoxide; NO_2_: nitrogen dioxide; O_3_: ozone; PM_2.5_: fine particle; PM_10_: inhalable particle; SO_2_: sulfur dioxide; SD: standardized deviation.

**Table 3:** Spearman correlation among air pollutants.

	CO	NO_2_	O_3_	PM_10_	PM_2.5_	SO_2_
CO	1.00	-	-	-	-	-
NO_2_	0.64	1.00	-	-	-	-
O_3_	0.17	0.21	1.00	-	-	-
PM_10_	0.59	0.69	0.56	1.00	-	-
PM_2.5_	0.63	0.67	0.53	0.95	1.00	-
SO_2_	0.37	0.56	0.34	0.63	0.57	1.00

*p* for all pairwise correlations < 0.05; CO: carbon monoxide; NO_2_: nitrogen dioxide; O_3_: ozone; PM_2.5_: fine particle; PM_10_: inhalable particle; SO_2_: sulfur dioxide.

**Table 4:** Percent change in odds of hospital admission for total and cause-specific stroke associated with each 10 µg/m^3^ increase in exposure to CO, NO_2_, PM_10_, PM_2.5_, SO_2_ and each 1 mg/m^3^ increase in exposure to CO.

	Percent Change in Odds, % (95% CI)		
	Total Stroke	Hemorrhagic Stroke	Ischemic Stroke
CO (mg/m^3^)	6.98 (2.61, 11.53)	4.61 (−5.01, 15.19)	8.49 (3.60, 13.61)
NO_2_ (µg/m^3^)	2.73 (2.21, 3.25)	4.14 (3.03, 5.27)	2.23 (1.66, 2.80)
O_3_ (µg/m^3^)	−0.03 (−0.09, 0.03)	−0.09 (−0.13, −0.06)	−0.12 (−0.19, −0.05)
PM_10_ (µg/m^3^)	1.26 (1.00, 1.52)	2.11 (1.50, 2.71)	1.06 (0.77, 1.35)
PM_2.5_ (µg/m^3^)	1.87 (1.37, 2.38)	2.96 (1.82, 4.13)	1.60 (1.04, 2.16)
SO_2_ (µg/m^3^)	24.89 (20.87, 29.06)	32.38 (23.72, 41.65)	21.97 (17.59, 26.52)

CO: carbon monoxide; NO_2_: nitrogen dioxide; O_3_: ozone; PM_2.5_: fine particle; PM_10_: inhalable particle; SO_2_: sulfur dioxide.

**Table 5:** Percent change in odds of hospital admission for stroke associated with each 10 µg/m^3^ increase in exposure to CO, NO_2_, PM_10_, PM_2.5_, SO_2_ and each 1 mg/m^3^ increase in exposure to CO, stratified by sex, age and season.

	Percent Change in Odds, % (95% CI)		
	Sex, Male	Sex, Female	*p*
CO	12.47 (6.55, 18.73)	−0.37 (−6.63, 6.30)	<0.01
NO_2_	2.65 (2.00, 3.31)	2.69 (1.88, 3.50)	0.95
O_3_	0.00 (−0.08, 0.08)	−0.06 (−0.13, 0.01)	0.28
PM_10_	1.03 (0.70, 1.37)	1.57 (1.16, 1.98)	0.05
PM_2.5_	1.98 (1.33, 2.63)	1.67 (0.89, 2.46)	0.55
SO_2_	20.65 (15.85, 25.66)	29.80 (23.28, 36.65)	0.03
	Age, <65 year	Age, ≥65 year	*p*
CO	10.04 (3.79, 16.67)	3.92 (−1.89, 10.07)	0.17
NO_2_	2.16 (1.42, 2.90)	3.25 (2.54, 3.97)	0.04
O_3_	0.02 (−0.07, 0.11)	−0.05 (−0.11, 0.01)	0.23
PM_10_	0.78 (0.41, 1.16)	1.71 (1.34, 2.07)	<0.01
PM_2.5_	1.24 (0.52, 1.96)	2.49 (1.79, 3.19)	0.01
SO_2_	9.84 (4.69, 15.24)	39.43 (33.44, 45.69)	<0.01
	Season, Warm	Season, Cold	*p*
CO	−3.93 (−10.24, 2.83)	14.92 (9.31, 20.82)	<0.01
NO_2_	−0.10 (−0.95, 0.75)	3.64 (3.11, 4.18)	<0.01
O_3_	−0.01 (−0.09, 0.06)	−0.08 (−0.19, 0.03)	0.32
PM_10_	0.08 (−0.16, 0.33)	1.97 (1.65, 2.30)	<0.01
PM_2.5_	0.32 (−0.47, 1.12)	2.85 (2.25, 3.46)	<0.01
SO_2_	−3.72 (−9.49, 2.41)	37.71 (32.59, 43.03)	<0.01

CO: carbon monoxide; NO_2_: nitrogen dioxide; O_3_: ozone; PM_2.5_: fine particle; PM_10_: inhalable particle; SO_2_: sulfur dioxide. CI: confidence interval.

**Table 6:** Percent change in odds of hospital admission for total and cause-specific stroke associated with each 10 µg/m^3^ increase in exposure to CO, NO_2_, PM_10_, PM_2.5_, SO_2_ and each 1 mg/m^3^ increase in exposure to CO, estimated by single- and 2-pollutant models.

	Percent Change in Odds, % (95% CI)		
	Total stroke	Hemorrhagic stroke	Ischemic stroke
CO (mg/m^3^)	6.98 (2.61, 11.53)	4.61 (−5.01, 15.19)	8.49 (3.60, 13.61)
+PM_2.5_	0.10 (−4.42, 4.84)	−5.75 (−15.21, 4.77)	2.72 (−2.42, 8.13)
+PM_10_	−0.12 (−4.48, 4.43)	−6.14 (−15.28, 3.97)	2.43 (−2.51, 7.62)
+SO_2_	4.56 (0.27, 9.03)	2.10 (−7.33, 12.47)	6.02 (1.21, 11.05)
+NO_2_	−3.63 (−8.04, 0.99)	−11.33 (−20.33, −1.31)	−0.29 (−5.34, 5.03)
+O_3_	7.80 (3.30, 12.49)	−0.52 (−9.81, 9.73)	10.88 (5.76, 16.25)
NO_2_ (µg/m^3^)	2.73 (2.21, 3.25)	4.14 (3.03, 5.27)	2.23 (1.66, 2.80)
+PM_2.5_	2.49 (1.87, 3.12)	3.83 (2.50, 5.18)	1.98 (1.29, 2.67)
+PM_10_	1.98 (1.35, 2.62)	2.88 (1.53, 4.25)	1.59 (0.89, 2.29)
+SO_2_	1.24 (0.66, 1.83)	2.32 (1.06, 3.59)	0.88 (0.23, 1.53)
+CO	2.91 (2.33, 3.50)	4.68 (3.44, 5.93)	2.23 (1.59, 2.88)
+O_3_	3.15 (2.60, 3.70)	3.47 (2.31, 4.64)	2.86 (2.26, 3.47)
O_3_ (µg/m^3^)	−0.03 (−0.09, 0.03)	−0.09 (−0.13, −0.06)	−0.12 (−0.19, −0.05)
+PM_2.5_	−0.31 (−0.39, −0.22)	−0.07 (−0.11, −0.03)	−0.42 (−0.51, −0.33)
+PM_10_	−0.42 (−0.50, −0.33)	−0.03 (−0.08, 0.01)	−0.51 (−0.60, −0.42)
+SO_2_	−0.29 (−0.36, −0.22)	−0.04 (−0.07, 0.00)	−0.37 (−0.45, −0.29)
+CO	−0.05 (−0.12, 0.01)	−0.09 (−0.13, −0.06)	−0.15 (−0.22, −0.08)
+NO_2_	−0.15 (−0.22, −0.09)	−0.06 (−0.10, −0.03)	−0.23 (−0.30, −0.15)
PM_10_ (µg/m^3^)	1.26 (1.00, 1.52)	2.11 (1.50, 2.71)	1.06 (0.77, 1.35)
+SO_2_	0.07 (−0.28, 0.42)	0.65 (−0.16, 1.48)	−0.04 (−0.43, 0.35)
+NO_2_	0.67 (0.35, 0.99)	1.17 (0.44, 1.91)	0.58 (0.23, 0.94)
+CO	1.23 (0.95, 1.51)	2.15 (1.51, 2.80)	0.99 (0.68, 1.30)
+O_3_	2.41 (2.06, 2.77)	1.72 (0.93, 2.53)	2.48 (2.09, 2.87)
PM_2.5_ (µg/m^3^)	1.87 (1.37, 2.38)	2.96 (1.82, 4.13)	1.60 (1.04, 2.16)
+SO_2_	−0.49 (−1.11, 0.15)	−0.35 (−1.80, 1.12)	−0.49 (−1.19, 0.21)
+NO_2_	0.51 (−0.09, 1.11)	0.71 (−0.63, 2.08)	0.51 (−0.15, 1.19)
+CO	1.83 (1.27, 2.39)	3.15 (1.89, 4.43)	1.44 (0.82, 2.07)
+O_3_	3.45 (2.78, 4.12)	1.37 (−0.10, 2.85)	3.78 (3.04, 4.53)
SO_2_ (µg/m^3^)	24.89 (20.87, 29.06)	32.38 (23.72, 41.65)	21.97 (17.59, 26.52)
+PM_2.5_	27.24 (22.01, 32.69)	34.05 (22.75, 46.40)	24.31 (18.64, 30.24)
+PM_10_	24.12 (18.72, 29.77)	25.82 (14.65, 38.08)	22.34 (16.43, 28.56)
+NO_2_	20.11 (15.66, 24.72)	23.31 (14.07, 33.29)	18.62 (13.73, 23.71)
+CO	23.90 (19.87, 28.06)	31.05 (22.43, 40.28)	21.05 (16.67, 25.60)
+O_3_	33.66 (28.82, 38.68)	27.81 (18.37, 37.99)	32.93 (27.57, 38.51)

All *p*-values were <0.05 and estimated using the likelihood ratio test. CO: carbon monoxide; NO_2_: nitrogen dioxide; O_3_: ozone; PM_2.5_: fine particle; PM_10_: inhalable particle; SO_2_: sulfur dioxide.

## Data Availability

The meteorological data are available at [http://data.cma.cn]. The air pollution data (CHAP dataset) are available at [https://weijing-rs.github.io; accessed on 15 October 2024]. The admission data are not publicly available.

## Abbreviations

The following abbreviations are used in this manuscript:

DLM	Distributed lag model
DALYs	Disability-adjusted life-years
NCDs	Non-communicable disorders
CO	Carbon monoxide
SO_2_	Sulfur dioxide
NO_2_	Nitrogen dioxide
PM_2.5_	Fine particle
PM_10_	Inhalable particle
O_3_	Ozone
ICD-10	International Statistical Classification of Diseases and Related Health Problems, 10th Revision
CHAP	China high air pollutant
R^2^	Cross-validated coefficient of determination
HI	Heat index
SD	Standard deviation
CI	Confidence interval
*df*	Degrees of freedom
DLNMs	Distributed lag non-linear models
ANS	Autonomic nervous system

## Appendix A

**Table A1:** Percent change in odds from total and cause-specific stroke associated with lag 0–3 days exposure to air pollution.

	Percent Change in Odds, % (95% CI)		
	Total stroke	Hemorrhagic stroke	Ischemic stroke
CO (mg/m^3^)			
Low	Ref.	Ref.	Ref.
High	−3.83 (−5.35, −2.28)	−1.83 (−5.46, 1.93)	−3.24 (−4.94, −1.52)
NO_2_ (µg/m^3^)			
Low	Ref.	Ref.	Ref.
High	2.61 (1.07, 4.18)	4.01 (0.33, 7.81)	1.75 (0.06, 3.47)
O_3_ (µg/m^3^)			
Low	Ref.	Ref.	Ref.
High	−1.15 (−2.44, 0.15)	6.12 (2.86, 9.48)	−2.71 (−4.12, −1.28)
PM_10_ (µg/m^3^)			
Low	Ref.	Ref.	Ref.
High	1.34 (−0.21, 2.92)	5.89 (2.15, 9.77)	0.83 (−0.88, 2.58)
PM_2.5_ (µg/m^3^)			
Low	Ref.	Ref.	Ref.
High	−0.81 (−2.39, 0.79)	3.98 (0.14, 7.96)	−1.75 (−3.48, 0.01)
SO_2_ (µg/m^3^)			
Low	Ref.	Ref.	Ref.
High	2.46 (0.85, 4.09)	5.91 (2.07, 9.90)	1.45 (−0.33, 3.25)

Low refers to pollutant concentrations below the median; High refers to pollutant concentrations greater than or equal to the median. CO: carbon monoxide; NO_2_: nitrogen dioxide; O_3_: ozone; PM_2.5_: fine particle; PM_10_: inhalable particle; SO_2_: sulfur dioxide.
